# Dataset Construction from Naturalistic Driving in Roundabouts

**DOI:** 10.3390/s20247151

**Published:** 2020-12-13

**Authors:** Laura García Cuenca, Carlos Guindel, Nourdine Aliane, José María Armingol, Javier Fernández Andrés

**Affiliations:** 1 Science, Computing and Technologies, Universidad Europea de Madrid, Calle Tajo s/n, Villaviciosa de Odon, 28670 Madrid, Spain; 2 Intelligent Systems Lab (LSI), Universidad Carlos III de Madrid, Av. Universidad 30, 28911 Leganés, Spain; cguindel@ing.uc3m.es (C.G.); armingol@ing.uc3m.es (J.M.A.); 3 Industrial System and Aerospace Engineering, Universidad Europea de Madrid, Calle Tajo s/n, Villaviciosa de Odon, 28670 Madrid, Spain; nourdine.aliane@universidadeuropea.es (N.A.); javier.fernandez@universidadeuropea.es (J.F.A.)

**Keywords:** naturalistic driving, data mining, roundabout maneuvers, video and images processing, dataset

## Abstract

A proper driver characterization in complex environments using computational techniques depends on the richness and variety of data obtained from naturalistic driving. The present article proposes the construction of a dataset from naturalistic driving specific to maneuvers in roundabouts and makes it open and available to the scientific community for performing their own studies. The dataset is a combination of data gathered from on-board instrumentation and data obtained from the post-processing of maps as well as recorded videos. The approach proposed in this paper consists of handling roundabouts as a stretch of road that includes 100 m before the entrance, the internal part, and 100 m after the exit. This stretch of road is then spatially sampled in small sections to which data are associated.

## 1. Introduction

An ideal autonomous vehicle is one driven by an expert system that behaves like a human driver would [1]. Automated driving implements a driver model transforming information perceived from real-world sensor measurements into actions on the vehicle’s actuators, such as steering wheel or pedals [2]. However, autonomous driving is a challenging task, and it is even more complex in dynamic environments, such as roundabouts or intersections, among others [3,4,5,6,7].

The status of the vehicle’s environment is undoubtedly one of the most significant sources for decision-making. In this aspect, environment perception from on-board sensors is an active field of research in this area. Over the last decade, the advent of deep learning techniques has meant an enormous leap forward in the performance of applications such as road user detection in sensor data [8]. High-performance deep-learning detection architectures, e.g., YOLO [9] or Faster R-CNN [10], have proven to be suitable for extracting reliable information from on-board camera images, even in complex traffic situations. The degree of sophistication exhibited by these algorithms has brought attention to the concept of weak supervision, in which imperfect data, such as the one obtained as a result from a deep neural network, is used to train other models without a relevant drop in their capabilities. Thus, recent works have shown the feasibility of automatically generating labels for different tasks such as lane marking recognition [11] or drivable path estimation [12]. In this work, an automatic image-processing pipeline is used to generate a high-quality description of the environment of the instrumented vehicle that includes all the relevant road users that must be considered for navigation.

Driver behavior modeling, specifically in complex scenarios, is also an active field of research during the last years, and several works can be found in the scientific literature addressing different issues. For example, a driver’s model in simulated environments is studied in [13], or the study in [14] proposes an approach based on the measured distance between vehicles, or in [4] that proposes the analysis of evasive maneuvers at intersections. Traffic flow of autonomous vehicles and conventional vehicles in shared traffic scenarios are also studied [15]. Computational and data mining techniques have also been applied to characterize drivers by exploiting data from naturalistic driving [16]. The study carried out in [17] uses data of more than 300 intersections. Different machine learning techniques are compared in the context of lane-changing behavior performed by humans in a semi-naturalistic and simulated environment [18]. These techniques are also applied in [19,20] to build models of autonomous vehicle trajectories in roundabouts and indirect maneuvers through naturalistic driving.

A proper driver characterization in complex environments using computational techniques depends ultimately on the richness and variety of the data obtained from naturalistic driving. In this sense, several works [21,22,23,24,25,26] propose merging several data sources obtained from smartphones, on-board sensors, and a specific data recording system. However, most proposed studies use private and not shared data, and their corresponding studies are hardly replicated.

Instead, most publicly available datasets aimed at autonomous driving focus on environment perception, such as the widely-used KITTI dataset [27] or the newest Waymo Open Dataset [28]; in both cases, the information available about the status of the vehicle is limited to its GPS location. Some recent datasets have included additional data to enable further low-level control research. For instance, nuScenes [29] is endowed with CAN bus data, including accelerations, torque, steering angles, and wheel speeds. Similarly, A2D2 [30] also includes vehicle bus data that complements the GPS/IMU information. Nevertheless, these datasets are still built with the requirements of on-board perception systems in mind, which makes them mostly impractical for modeling maneuvers in specific environments.

This article proposes the construction of a dataset from naturalistic driving specific for roundabouts maneuvers. The approach proposed in this paper consists of handling roundabouts as a stretch of road, including the 100 m before, the inner part, and the 100 m after. Each roundabout is divided into several sections to which data are associated. The goal of the paper is to make the dataset open and available to the scientific community for performing their own studies.

The rest of this paper is organized as follows. Section 2 presents the on-board setup used for data acquisition. Section 3 presents the approach used for roundabout segmentation and explains the process of obtaining the main variables of the dataset and its organization. Section 4 ends the paper by drawing some conclusions and comments about the final dataset.

## 2. Experimental Setup for Raw Data Collection

To capture the state of the car, the driver’s behavior as well as the environment during naturalistic driving, an experimental data acquisition system has been developed. The ad-hoc experimental system is based on two parts: A smartphone and an Arduino board.

The smartphone, located in the dashboard, was used for recording videos of the driving scenes with its rear camera at a rate of 30 fps, which was enough as a vision-based perception system. It featured a 12 MP, 1/2.6” CMOS sensor (with 1.4 µm pixels) and an F/1.7 lens with 4.2 mm of focal length. This combination yielded an HFOV of around 65°. The smartphone was also used for capturing the vehicle’s GPS locations as well as its different accelerations.

The Arduino board was used to capture driver interaction with its vehicle, namely, signals related to brake, clutch, and acceleration pedals, vehicle’s right and left blinkers, and the steering wheel rotation obtained from a 7-bits absolute encoder mounted on the steering shaft. The full range of the steering wheel is about three complete revolutions (−540° to 540°), giving a resolution of 1.4° of steering wheel per bit. Furthermore, the hardware provided 2 extra buttons for labeling up to 4 situations that could be interesting to mark during the route, for instance, the start and the end of a roundabout area. The sampling rate was set to 2 Hz, which was enough for automotive navigation and driver interaction with the vehicle. The Arduino was directly powered by the vehicle’s battery through its DC barrel plug. As the standard Arduino mega board had its built-in voltage regulator, an input voltage range of 7 to 12 volts was guaranteed, and no additional electronics were required to overcome overvoltage transient. The different sensors were connected to the Arduino through voltage dividers reducing the 8 V in the sensor’s outputs to 5 V. The smartphone and the Arduino board were connected through a Bluetooth connection.

**Figure 1:**
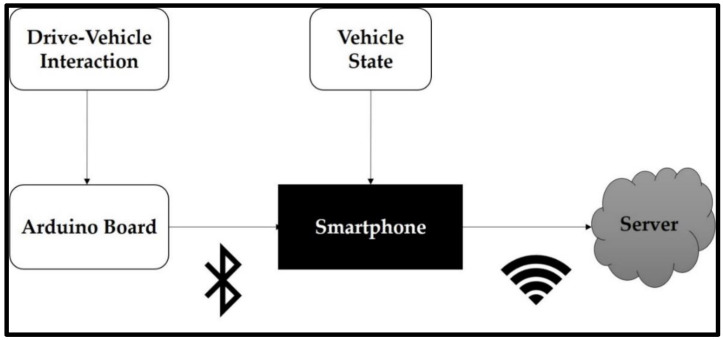
Experimental setup for raw data collection.

To handle the collected data properly, an Android app provided users with some functionalities, such as driver identification, enabling video recordings, start or stop data recording, and uploading the recorded data to a server at the end of each trip through a WIFI connection. The logical organization of the experimental setup is depicted in Figure 1.

**Figure 2:**
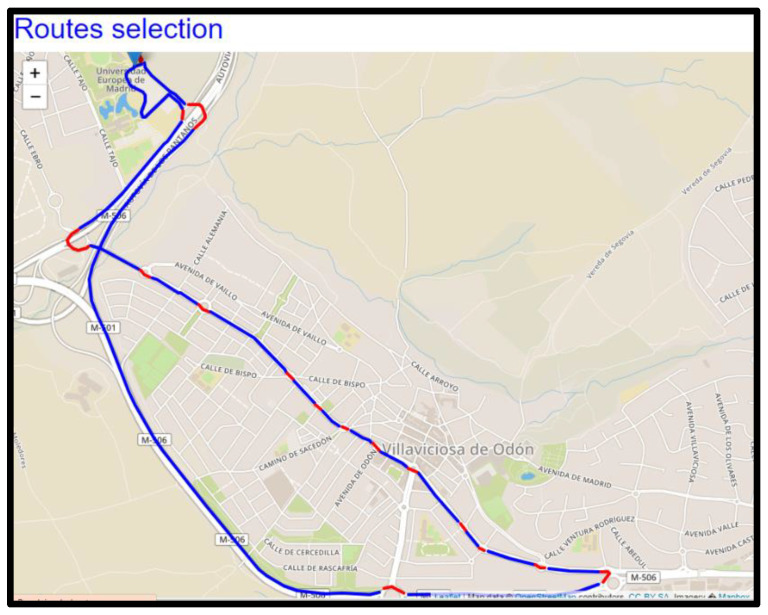
A snapshot showing a path and a route with its roundabouts marked in red.

The server, located in the authors’ institution, was used for data repository and provided an application for route viewer. This application was used to filter and search for specific data by routes or drivers and to visualize and explore temporal and spatial information of the recorded trips by just moving the mouse around roundabouts on the map. A snapshot of such visualization is shown in Figure 2.

In order to collect driving data for roundabout maneuvers through the hardware setup defined in this section, the raw data of the different controls of the vehicle used for the experimentation were obtained, such as pedals, flashing lights, steering wheel turn, and other buttons, which will later be integrated into a single connector accessible from the trunk. These sensors will be connected to a data acquisition module based on an Arduino board whose main objective was to read the state of the sensors in the loop, compose a data frame, and send it via Bluetooth to the smartphone application (APP) developed ad-hoc to measure the information provided by smartphone sensors such as GPS (Global Positioning System), accelerometers, a gyroscope, and a camera.

All this information was collected from the vehicle by the data acquisition system, which will be pre-processed continuously, generating a data frame ordered by time (timestamp) that, together with the videos recorded in real-time of the different routes, were stored temporarily on the phone until it was forwarded to the web server. This server centralized the data of different controllers and processed it until its final storage form in a database designed in MySQL.

Besides the collected raw data in real-time by the on-board experimental setup, several aggregated parameters were generated from post-processing of the routes and videos, as explained in Section 3.

## 3. Data Mining Process and Dataset Organization

The components of the dataset come from three sources: Processing collected data from the on-board instrumentation, data from obtained cartography, and data from recorded videos. The on-board instrumentation provided information in terms of vehicle GPS positions, vehicle speed, and steering wheel turns. The information obtained from the off-line processing of cartography was related to roundabouts diameters and number of lanes, the assignment of labels to the different segments. Finally, the off-line processing of the recorded videos provided the dataset with information related to the agents in the vehicle’s vicinity, depicting the traffic situation. In this aspect, the set of parameters extracted from the videos included crowdedness, a variable measuring the number of dynamic objects in the traffic scene; the presence of or not of obstacles, such as vulnerable road user (pedestrians, cyclists, and motorcyclists); the distance to the closest vehicle in the current lane; and their dominant orientation.

### 3.1. Data Acquisition Process

Several drivers participated in the data acquisition process by driving on several roads in the metropolitan area of Madrid, Spain, during a period of different months, including routes with roundabouts with different diameters and with single and multiple lanes. All the drivers used the same vehicle equipped with a smartphone running the APP, as described previously in Section 2.

In order to address a decisive selection of data and extract the most information and knowledge from the different sources analyzed, a real experiment was carried out on a test route. From this experimentation, data could be collected to establish an appropriate selection that, in the first instance, helped us determine a final set of data to be able to obtain human driving patterns in roundabouts and model the autonomous driving expert system. The experimentation was prepared to specify which elements or components would be tested, thus that the process of validation and verification of the necessary sources and data could be carried out. Besides, through this test plan, information on errors, defects, or failures could be obtained, thus making possible the pertinent corrections, as appropriate, and ensuring the quality of the data collected as sources.

For example, the experimentation route, which lasted 30 min and passed through 12 roundabouts, provided the data set defined in Table 1, having been created, defined, generated, and processed based on the information from the data acquisition system developed for data acquisition in the present research work and summarized in Section 2.

After reviewing these data obtained from the hardware setup, it was necessary to add more data to be processed together with those already obtained from the acquisition system to obtain precise information on roundabouts as specified in the next section.

### 3.2. Roundabout Segmentation

**Figure 3:**
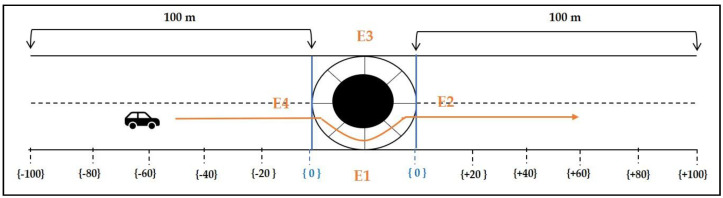
Points of interest (or labels) corresponding to a vehicle undertaking E2 exit.

When approaching a roundabout, the behavior of a driver, from an attention point of view, can be divided into three phases: Namely, approaching the roundabout, maneuvering inside it, and leaving the roundabout. Thus, to capture the essence of these maneuvers, roundabouts were divided into three sections called “before,” “inner,” and “after.” The “before” and “after” sections of 100 m each were divided into segments of 20 m. These segments were labeled in the dataset as positions corresponding to {−100, −80, −60, −40, −20} before entrance, and {+100, +80, +60, +40, +20} after exiting. Regarding the “inner” section, it was divided into portions of 45° taking as references the roundabout center and entry points. The corresponding points inside the roundabout were labeled as {0°, 45°, 90°, 135°, 180°, etc.}. The number of labels within the inner section depends on the exit (E1, E2, E3, or E4) undertaken by the vehicle. Figure 3 shows the points of interest (or labels) corresponding to a vehicle undertaking E2 exit.

### 3.3. Feature Engineering

**Figure 4:**
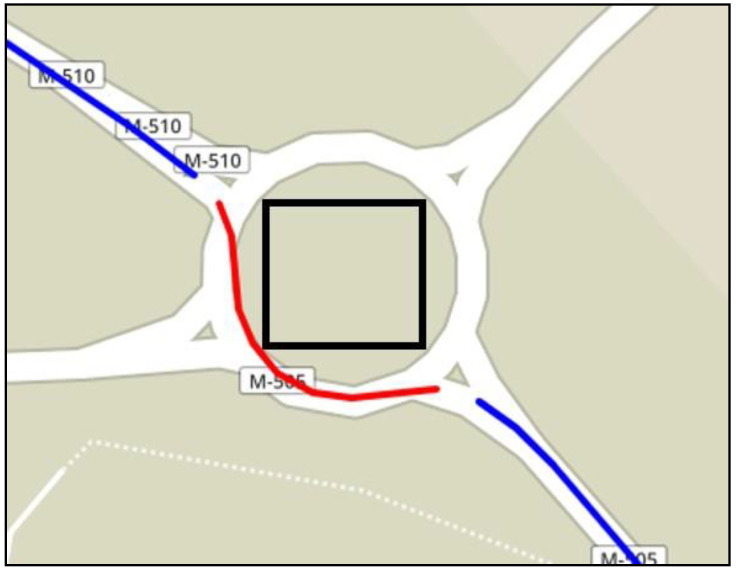
The stretch road, showing the point of interest of a roundabout.

The starting point of the dataset building was the selection of roundabouts of a specific route on the map, which was carried out with a route viewer used over OpenStreetMaps. This was done by drawing a square envelope around roundabouts for performing queries and using appropriate functions of the OpenStreetMaps API [31] that returned roundabout attributes, such as diameter, number of lines, and the GPS of the roundabout center. The square was then moved along the routes for processing roundabouts, as depicted in Figure 4. In the same way, a specific query OpenStreetMaps API function was used to isolate the entry, and the exit points of the roundabout, which were then used as a reference to label the different segments, as explained in the previous section.

**Figure 5:**
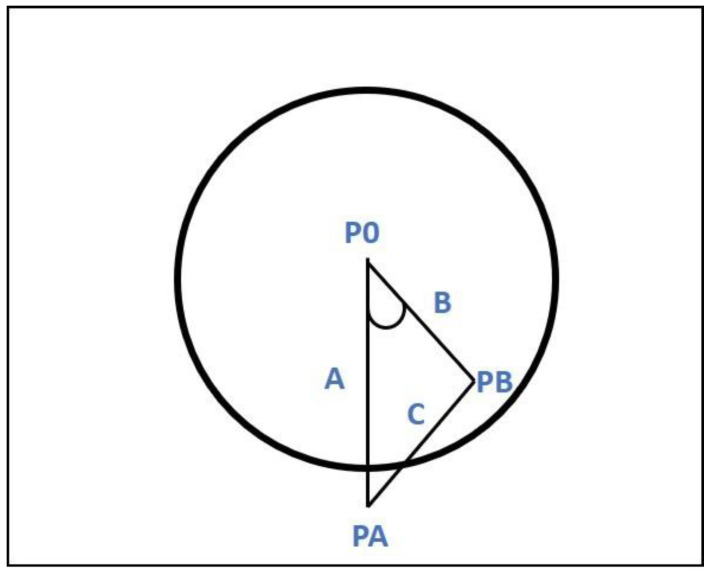
Segmentation in the 45-degree angle section within the roundabout.

The labeling of inside roundabout segments was carried out using the roundabout entry point and its geometrical center as reference points, as depicted in Figure 5. The labels were obtained starting from the entry point and moving along the GPS locations, and then the cosine rule was used as a trigger to detect points corresponding to sections of 45°. Given a triangle (PO, PA, PB), where PA was the entry point, PO was the roundabout center, PB was a moving point, and A, B, and C were the corresponding triangle sides. A section of 45° corresponded to a distance C when the following condition is fulfilled:

(1) $$C^{2}\geq A^{2}+B^{2}-\sqrt{2}AB$$

Although the raw data were collected at a frequency of 2 Hz, variables in the dataset were only associated with the labels of roundabouts. Thus, after discarding samples corresponding to speed in congested traffic, the vehicle speed and the steering angle were calculated by averaging the collected data over each segment.

### 3.4. Video and Image Processing

As video and image processing was concerned, the recorded videos were synchronized with the GPS data, making it possible to extract the set of images corresponding to the roundabout’s labels, as defined in the previous section. For each segment, the GPS timestamps at the entry and exit points were determined, and then all video frames captured during that interval were assigned to the corresponding segment. In practice, this time interval was increased by a small margin (about 500 ms) at both ends; in that way, those frames captured in between consecutive segments were considered as part of both, enlarging the set of frames available for parameter extraction and neutralizing the effect of any eventual minor desynchronization.

In order to identify the road users in the traffic scene, each video frame goes through an image-processing pipeline whose central part is a deep neural network based on the Faster R-CNN paradigm [32]. Faster R-CNN is a widely used object detection architecture that works in two well-differentiated stages: The first one, made of a lightweight Region Proposal Network (RPN), is used to find image regions that are likely to contain objects, whereas the second one is aimed at classifying and refining those proposals. Both parts employ the same set of features, learned from labeled samples through a multi-task loss that considers all the tasks involved in the procedure. As a result, a list of objects, represented as bounding boxes in image coordinates, is obtained. Each is endowed with an estimate of the type of object (e.g., car or pedestrian) and a score measuring the classification confidence.

In this work, the variant of Faster R-CNN presented in [33] was used, which was endowed with an additional inference branch to estimate the orientation of the objects with respect to the camera (i.e., their observation angle or viewpoint). This task was posed as a classification among discrete viewpoint bins, which has been proven adequate for on-board perception systems. The new branch exploits the same set of features used for detection and classification, thus introducing a negligible overhead. To maximize the accuracy of the results, the model employed in this work uses a ResNeXt-101 feature extractor with a 32 × 8d template [34], which features an increased capacity compared to the typical ResNet models. A Feature Pyramid Network scheme is used for feature extraction.

The model has been trained on the nuScenes dataset [29], a large-scale dataset that is lately gaining popularity in the on-board perception field. Unlike other possible alternatives, nuScenes features 3D object labels, enabling the training of the viewpoint-enabled Faster R-CNN variant employed in this work. Thus, the 2D bounding box annotations used for training are obtained as the projection of the 3D cuboid labels onto the image, including the observation angle as an additional field. Only the dynamic categories included in the nuScenes detection challenge (i.e., car, pedestrian, bicycle, bus, construction vehicle, motorcycle, trailer, and truck) are considered. The final model has been obtained by fine-tuning COCO pre-trained weights during 100 k iterations. Apart from the customary horizontal flipping, scale jittering (from −10% to 15% of the original scale) has been applied to training images to improve the robustness of the resulting model. Additionally, the repeat factor sampling proposed in [35] is used to mitigate the effects of class imbalance. The resulting deep neural network model is aimed to provide 2D bounding box detections of the objects. Frames are fed into the model at full scale during inference to achieve optimal detection performance. Later, objects classified with a score lower than 0.8 are removed to avoid false positives. At this point, it should be noted that detections are localized within the 2D image coordinates, whereas the variables to be obtained at the end of the image-processing pipeline rely on the objects’ 3D position. In order to retrieve the objects’ 3D location, a simple distance estimation based on the pinhole camera model is used. As will be shown later, this approach has been found enough for roundabout scene awareness. Given that the camera’s focal length is known, the depth of an object with respect to the camera, in meters, can be computed by assuming an estimate of the real dimensions of that object; in this case:

(2) $$Z=\frac{fH}{h}$$

where f is the focal length in pixels, H is the real height (in meters) of the object, and h is the height in pixels of its representation in the image. Height estimates are obtained from a set of predefined values, one per category, representing the average height of all the 3D labels in the nuScenes dataset belonging to that category. Once the depth coordinate is available, the lateral location can be straightforwardly derived, and thus, each object can be fully localized in the camera’s local coordinate frame.

With this information, objects whose depth coordinate was larger than 30 m were filtered out to avoid considering them in the computation of the variables. Then, each instance was assigned to one of three regions in which the image was divided (i.e., left, center, and right) according to the position of the center of its 2D bounding box. Once the objects in a frame were fully identified, the value of the four traffic-related parameters corresponding to that moment could be computed. As the dataset was organized into roundabout segments, and several video frames were available for each of them, single-frame estimates were subsequently aggregated to represent the overall status of the environment along each segment. The processing was carried out separately for each image region as follows:

- ‘Crowdedness’ is obtained as the count of all traffic participants detected in each frame. This count is later averaged across all the frames and rounded to the nearest integer.
- ‘Presence of VRUs’ considers if any of the filtered detections belongs to the pedestrian, bicycle, or motorcycle categories. A segment is flagged when VRUs are present in more than 50% of its frames, which are usually deemed vulnerable road users (VRUs).
- ‘Distance to the closest vehicle’ is the minimum depth coordinate among all the detected vehicles. Here, only the car, bus, construction vehicle, trailer, and truck categories are considered. The single-frame distance estimates in a segment are ultimately summarized by their mean value.
- ‘Dominant orientation’ is obtained in several steps. Firstly, the object’s yaw angle is derived using both the viewpoint estimate given by the neural network and the location computed through the pinhole camera model, as in [35]. Afterward, following the approach employed for viewpoint estimation, the 360° range of possible yaw angles is discretized into eight bins, and the bin representing the orientation of the closest object becomes the value of the “dominant orientation” parameter. Finally, the value describing a segment is the most frequent (i.e., the mode) among the successive estimates.

#### Traffic Parameters Quality Assessment

We conducted some experiments to assess the validity of the approach using the nuScenes dataset. To that end, we followed the official train/validation splits: The first was used to train the network and define the prototypical height for each object category, whereas the second was used to provide quantitative results.

We compared the results obtained by the proposed processing pipeline for each of the four variables of interest with the ground-truth values given by the dataset 3D labels. Note that the restrictions used to build the dataset were also imposed here; i.e., only objects closer than 30 m and belonging to a dynamic category are considered.

The ‘Crowdedness,’ ‘Distance to the closest vehicle,’ and ‘Dominant orientation’ variables are analyzed in Table 2 employing the mean absolute error (MAE) and the root-mean-square error (RMSE). As shown by the results, which were provided separately for each image region, the average error was well below 1 unit in the estimation of both the number of agents and the dominant orientation bin, which confirms the high accuracy of the detection and viewpoint estimation framework. The error in the estimation of the distance to the closest vehicle is heavily affected by the presence of outliers, but they are mostly smoothed out during the construction of the dataset due to the aggregation of several video frames. Despite this, the median absolute error is as low as 1.74 m, which should be enough for spatial reasoning.

It is noteworthy that the ‘Distance to the closest vehicle’ variable implicitly conveys binary information about the presence of vehicles in the 30 m area in front of the ego-car. The error reported above only considers true positive instances; however, precision and recall values for the binary classification problem can be found in Table 3, showing the high reliability of the estimate. Table 3 also shows the precision and recall of ‘Presence of VRUs’, which is itself a binary variable. The results show that the selected score threshold leads to high precision in the detection of VRUs; on the other hand, recall values, diminished by the occurrence of false negatives, are less critical as they will largely benefit from the use of consecutive video frames in the computation of the final values.

**Figure 6:**
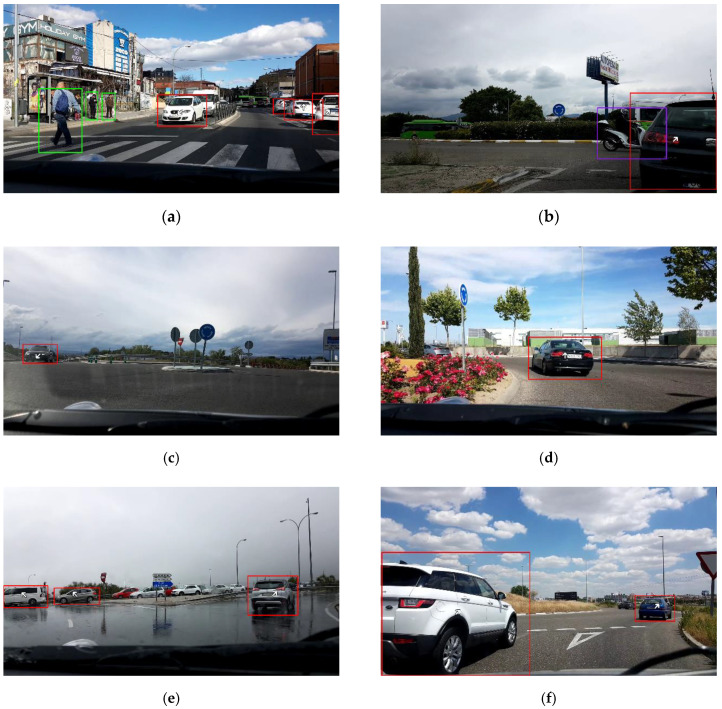
Snapshots of the image-processing pipeline on four video frames taken from the dataset. Only detections fulfilling score and distance criteria are depicted. (**a**) explanation; (**b**) explanation; (**c**) explanation; (**d**) explanation; (**e**) explanation; (**f**) explanation.

For qualitative assessment, Figure 6 depicts the result of the image processing pipeline on four video frames taken from the dataset, where relevant objects are indicated by bounding boxes and categorized by color, namely: Red for cars, green for pedestrians, and purple for motorcycles. Viewpoint estimates for vehicles are also included as white arrows in the center of their bounding boxes. Table 4 shows some estimated parameters from the images of Figure 6.

### 3.5. Dataset Organization

The final dataset is embedded in a single CSV file, and it is organized in three levels; namely, the dataset can be seen as a group of routes, where each route is a collection of roundabouts, and each roundabout is divided into segments defined as points of interest. Each segment has several attributes, namely vehicle speed, steering angle, roundabout diameter, number of lines, and data generated from video processing, which are crowdedness, defined as the number of detected objects in each frame, presence of vulnerable users, the distance to the closest vehicle and its dominant orientation. Variables obtained from video processing are searched for in three separate regions (left, center, right) of each frame.

The final dataset consists of 33 routes containing 337 roundabouts with their corresponding attributes filtered and prepared for high-level processing. The headings and the variable identifiers are shown in Table 5. The dataset can be downloaded from a GitHub repository at: https://github.com/Lauragcuenca/dataset-roundabout.

To draw a first picture of the dataset, a basic statistical analysis has been carried out. In this sense, the vehicle speed variable is between 0 and 69 km/h, with an average speed of 36.77 km/h. The diameter of roundabouts ranges from 13 m to a large one of 103 m, with an average value of 47.65 m. The most frequently repeated diameter was about 50 m, and most of the roundabouts were with two lanes. The steering angle ranges from −7.8° to +40°, with an average of 15.7°. The average value of crowdedness detected in each image region (i.e., left, center, and right) was 0.25, 0.26, and 0.39, respectively. The highest value was reached on the right side, where up to five different objects were identified in the same roundabout segment; for the other two areas, the value peaked at four. VRUs were present in the right region in around 0.4% of roundabout segments. This frequency dropped to 0.3% in the central area and 0.1% on the left side. On the other hand, the average distance to the closest vehicle was significantly higher in the central part of the image (20.05 m) than on the sides (16.62 m on the left, 16.83 m on the right). Distances spanned a range that went from 2.8 m to the upper bound, set to 30 m. Regarding the dominant orientation, all the values were integers between 0 and 7, representing each of the 8 possible 45° bins. Finally, as expected, the most frequent orientation in the left area was ‘backward’ (bin 6), whereas ‘forward’ (bin 2) was prevalent in the central and right regions.

## 4. Conclusions

A methodology for dataset construction from naturalistic driving specific to roundabouts maneuvers is proposed. The approach proposed in this paper consists of handling roundabouts as a stretch of road that includes the 100 m before the entrance, the inner part, and the 100 m after the roundabout exit, which then spatially sampled in several sections to which data are associated. The dataset components come from three sources. The first source consists of the processing of raw data collected from the on-board instrumentation to obtain the vehicle speed and the steering wheel angle. The second source is related to exploiting cartography and the recorded routes to obtain the roundabout diameters and their number of lanes as well as for generating the labels of the different roundabout’s sections. Finally, the off-line processing of the recorded videos permits to generate traffic data in the vehicle vicinity, namely crowdedness, defined as the number of dynamic objects, the presence or not of vulnerable road users, such as pedestrians, cyclists, or motorcyclists, the distance to the closest vehicle and the dominant orientation the vehicles in the surroundings.

The dataset is open and can be exploited by the scientific community to perform their own studies. Although datasets aimed at autonomous driving already exist, they generally provide more extensive data, including raw sensor data. In contrast, the dataset presented in this paper is intended to allow researchers to focus on this specific application of roundabout maneuvering, avoiding the need to apply complicated image processing algorithms at the beginning of the process to obtain meaningful information about the vehicle environment. The parameters included in the dataset already summarize substantial decision-making factors, in line with the “affordance” representation frequently used in the related literature [32,36].

The presented dataset may be helpful for generating knowledge using machine learning techniques carrying out driving pattern classification in roundabouts, and for predicting vehicle speed and steering wheel in the different sections of roundabouts, as shown in [19], where algorithms such as Support Vector Machine, Lineal Regression, and Deep Learning are used to obtain different predictive data models. Other machine learning techniques that can be used on this autonomous driving dataset are algorithms based on reinforcement learning as in [6], where a Markov decision process (MDP) was used to study the behavior of a vehicle in order to safely navigate roundabouts using the Q-learning algorithm in a simulation environment. Regarding future works, it is planned to upgrade the built dataset and to apply the same approach to generate similar datasets driving in the urban intersection and highway entrances.

## Figures and Tables

**Table 1:** Example of list of parameters for the data acquisition.

Data	Source	Data	Source
Timestamp	Smartphone	Seat_belt	Vehicle
Longitude	Smartphone	Clutch	Vehicle
Latitude	Smartphone	Brake	Vehicle
Steering angle	Vehicle	Throttle	Vehicle
Video	Smartphone	Speed	Smartphone
Left_blinker	Vehicle		Aggregated
Right_blinker	Vehicle		Aggregated

**Table 2:** Mean absolute error (MAE) and root-mean-square error (RMSE) of single-frame estimations on the nuScenes validation set for three of the parameters computed through the image processing pipeline.

	MAE				RMSE			
Parameter	Left	Center	Right	Total	Left	Center	Right	Total
Crowdedness	0.40	0.22	0.40	0.34	0.89	0.62	0.89	0.81
Distance to cl. vehicle	3.19 m	2.57 m	2.50 m	2.72 m	4.42 m	3.40 m	3.71 m	3.83 m
Dominant orientation	0.21	0.34	0.44	0.34	0.91	0.83	1.15	0.98

**Table 3:** Precision and recall (%) of single-frame estimations on the nuScenes validation set for the ‘Presence of VRUs’ parameter and the detection of the closest object.

	Precision				Recall			
Parameter	Left	Center	Right	Total	Left	Center	Right	Total
Closest veh. detection	90.6	86.8	92.4	89.9	84.1	96.3	87.9	89.4
Presence of VRUs	87.1	88.8	88.3	87.9	67.3	68.3	64.0	66.3

**Table 4:** Estimated values for traffic-related parameters from the video frames showed in Figure 6. The dominant orientation variable includes the yaw orientation bin as well as the direction represented as (FW: Forward, BW: Backward, L: Left, and R: Right, and combinations among them).

Frame	Region	Crowd.	Presence of VRUs	Distance to the Closest Vehicle (m)	Dominant Orientation
(a)	Left	3	Yes	-	-
	Center	1	No	14.3	7 (BW-L)
	Right	3	No	10.9	4 (R)
(b)	Left	0	No	-	-
	Center	0	No	-	-
	Right	2	Yes	4.7	3 (FW-R)
(c)	Left	1	No	24.0	6 (BW)
	Center	0	No	-	-
	Right	0	No	-	-
(d)	Left	0	No	-	-
	Center	1	No	10.7	2 (FW)
	Right	0	No	-	-
(e)	Left	2	No	20.7	0 (L)
	Center	0	No	-	-
	Right	1	No	11.6	3 (FW-R)
(f)	Left	1	No	3.7	3 (FW-R)
	Center	0	No	-	-
	Right	1	No	17.9	3 (FW-R)

**Table 5:** Dataset organization.

Label	Description
id_route	Identifier of route
id_roundabout	Identifier of roundabout
Segment	Data segment
segment_angle	Segment inner roundabout
Diameter	Diameter roundabout
Lanes	Lanes roundabout
Speed_vehicle	Speed vehicle in data segment
Steering_angle	Steering_angle in data segment
Crowdedness_left	Number of dynamic agents (left side)
Crowdedness_center	Number of dynamic agents (center)
Crowdedness_right	Number of dynamic agents (right side)
Vrus_left	0 = no VRUs, 1 = presence of VRUs (left side)
Vrus_center	0 = no VRUs, 1 = presence of VRUs (center)
Vrus_right	0 = no VRUs 1 = presence of VRUs (right side)
Distance_left	Distance to the closest vehicle (left side)
Distance_center	Distance to the closest vehicle (center)
Distance_right	Distance to the closest vehicle (right side)
Orientation_left	Dominant orientation bin (left side)
Orientation_center	Dominant orientation bin (center)
Orientation_right	Dominant orientation bin (right side)
